# Formulation and Evaluation of Fenbendazole Extended-Release Extrudes Processed by Hot-Melt Extrusion

**DOI:** 10.3390/polym14194188

**Published:** 2022-10-06

**Authors:** Gilberto S. N. Bezerra, Tielidy A. de M. de Lima, Declan M. Colbert, Joseph Geever, Luke Geever

**Affiliations:** PRISM Research Institute, Technological University of the Shannon: Midlands Midwest, University Road, N37HD68 Athlone, Ireland

**Keywords:** hot-melt extrusion, extended-release, fenbendazole

## Abstract

This study aimed to demonstrate the feasibility of hot-melt extrusion in the development of extended-release formulations of Fenbendazole (Fen) dispersed in PEO/PCL blend-based matrices. Their thermal, physical, chemical and viscosity properties were assessed by differential scanning calorimetry, thermogravimetric analysis/derivative thermogravimetry, Fourier transform infrared spectroscopy, X-ray diffraction spectroscopy, and melt flow index. Drug dispersion was analyzed by scanning electron microscopy with electron dispersive X-ray spectroscopy, and drug release was evaluated by ultraviolet-visible spectroscopy. A thermal analysis indicated the conversion of the drug to its amorphous state. FTIR analysis endorsed the thermal studies pointing to a decrease in the drug’s crystallinity with the establishment of intermolecular interactions. XRD analysis confirmed the amorphous nature of Fen. MFI test revealed that PCL acts as a plasticizer when melt-processed with PEO. SEM images displayed irregular surfaces with voids and pores, while EDX spectra demonstrated a homogeneous drug distribution throughout the polymeric carrier. Dissolution testing revealed that PCL retards the drug release proportionally to the content of such polymer incorporated. These melt-extruded matrices showed that the drug release rate in a PEO/PCL blend can easily be tailored by altering the ratio of PCL to address the issues related to the multiple-dosing regimen of Fen in ruminants.

## 1. Introduction

Fenbendazole (Fen) is a broad-spectrum anthelmintic used to treat gastrointestinal nematodes in ruminants (e.g., cattle, sheep and goats). Fen acts by binding to β-tubulin, inhibiting further polymerization of α- and β-tubulin subunits, disrupting microtubule function, and leading to a lethal effect. Due to its mechanism of action, optimum efficacy has been related to the time of parasite exposure to active drug concentrations and is only evidenced after successive doses [1]. Furthermore, formulations have been developed to improve Fen efficacy by prolonging its (i) administration by urea-molasses blocks [2] and (ii) release by intraruminal devices [3].

Fen has limited solubility in water, which affects its dissolution in the enteric fluid, absorption, and clinical efficacy. The literature states that the oral administration of an amorphous benzimidazole leads to a better dissolution rate and absorption when compared to those of its crystalline form, as well as only half a dose of the amorphous form can produce efficacy equivalent to that of its crystalline form [4]. Hot-melt extrusion (HME) has proven to be an efficient technique for the production of amorphous solid dispersions with improved bioavailability [5].

HME is a continuous manufacturing process that converts raw material into a product of uniform density and shape under controlled conditions [5]. The extensive mixing provided by a twin-screw extruder leads the active pharmaceutical ingredient (API) to interact with a polymeric matrix at a molecular level. Despite HME has many advantages over other techniques, there are still some challenges in the development of melt-extruded formulations [6], such as screening and selection of polymers based on their chemical and physical properties since these characteristics have to be in line with the API and the processing technique limitations [7]. Therefore, understanding the physicochemical properties of both the API and polymers is required when developing a solid dispersion since their properties after extrusion will dictate the formulations’ dissolution behavior [8].

Since the mid-1970s, biodegradable polymers have become vastly applied as carriers in the design of drug delivery systems [9]. Aliphatic polyesters are the most promising ones for extended-release formulations due to their capacity to degrade through the hydrolysis of the ester bond into non-toxic small molecular weight compounds that can be excreted by metabolic pathways [10]. A large number of these polymers are known for their specific and unique characteristics, so melt-blending them during extrusion is an efficient strategy to combine their desired properties without any chemical bonding [11].

Moreover, a balance of hydrophobicity and hydrophilicity is required to prevent recrystallization and control drug dissolution when processing solid dispersions by HME [12], so uncountable studies have been published blending polycaprolactone (PCL) and poly(ethylene oxide) (PEO) to the design of drug delivery systems, showing the versatility of PEO/PCL based-blending matrices and potential application in the development of new extended-release formulations. Grehan et al. [5] processed blends of PEO and PCL by HME to control the release of 4-acetamidophenol. Lyons et al. [11] also applied the melting extrusion process to produce monolithic matrices between PEO and PCL to deliver carvedilol. PEO is a semi-crystalline, hydrophilic, thermoplastic polymer capable of hydrating and swelling in contact with water, which makes PEO vastly applied in controlled-release tablets [13]. Since PEO processed by HME has proven to increase Fen dissolution properties demonstrating fast drug release rates [14], we hypothesized that blending PEO with a biodegradable aliphatic polyester as PCL, which is a highly hydrophobic and crystalline polymer able to degrade very slowly, would retard Fen release rates.

Fen can be prescribed as a single therapeutic dose or split over several days into multiple doses depending on the species [15]. During anthelmintic therapy, a consistent exposure of the worms to the drug is desired; nevertheless, divided doses can lead to sub-therapeutic concentrations of the anthelmintic increasing the risk of the selection of resistant parasites [16]. Hence, there is a need for extended-release formulations of Fen that can minimize not only the therapeutic problems associated with the multiple-dosing regimen in ruminants but also reduce the stress on animals due to repeated administration [17].

This study aims to demonstrate the feasibility of HME in the development of extended-release formulations of Fen dispersed in PEO/PCL blend-based matrices. Although twin-screw extruders have shown over the years to be one of the most efficient technologies to obtain higher mixing levels required for solid dispersions, the high thermal and mechanical energy applied during the extrusion process can affect the quality of the monolithic matrices; therefore, their thermal, physical, chemical, and viscosity properties were assessed using differential scanning calorimetry (DSC), thermogravimetric analysis (TGA)/derivative thermogravimetry (DTG), Fourier transform infrared spectroscopy (FTIR), X-ray diffraction spectroscopy (XRD), and melt flow index (MFI), drug dispersion was analyzed by scanning electron microscopy (SEM) with electron dispersive X-ray spectroscopy (EDX), and drug release was evaluated by ultraviolet-visible spectroscopy (UV-Vis).

## 2. Materials and Methods

### 2.1. Materials

**Figure 1:**
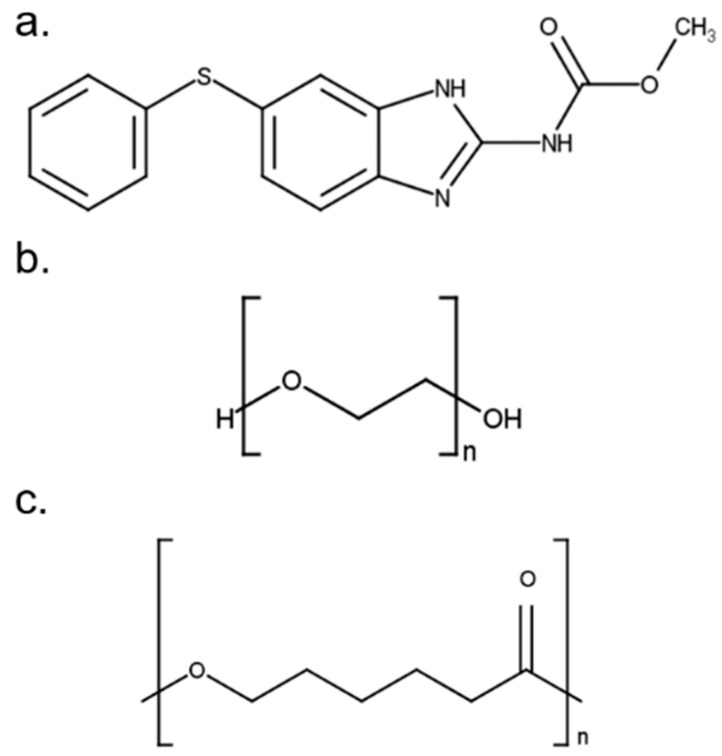
Chemical structures of (**a**) Fen, (**b**) PEO and (**c**) PCL designed using MarvinSketch 15.4.6.

Fen (methyl N-(6-phenylsulfanyl-1H-benzimidazol-2-yl)carbamate) was purchased from Molekula, PEO (*M_w_* 100,000–200,000 g mol^−1^) was obtained from Alroko^®^, and PCL (*M_w_* 50,000 g mol^−1^) was supplied by Perstorp. All other chemicals and reagents were of analytical reagent grade. The chemical structures of the API and polymers used in this study are shown in Figure 1.

### 2.2. Thermal Characterization by DSC and TGA/DTG

Calorimetric curves were obtained using a Pyris 6 DSC (Perkin Elmer) using between 6 and 8 mg of the sample in lid-sealed aluminum pans, under nitrogen atmosphere flow of 30 mL min^−1^, standard heat/cool/heat cycles of 30/100/30/300 °C in a rate of 10 °C min^−1^ for polymers and melt-extruded formulations, and standard heat from 30 to 300 °C at a rate of 10 °C min^−1^ for the API.

TGA curves were obtained using a Pyris 1 TGA (PerkinElmer). Samples were analysed using 10 mg of the sample in aluminium pans, under nitrogen atmosphere flow of 20 mL min^−1^, and standard heat from 30 to 700 °C at a rate of 10 °C min^−1^.

DSC and TGA/DTG measurements were performed using Pyris Manager Software.

### 2.3. Fourier Transform Infrared Spectroscopy

PerkinElmer Spectrum One with a universal ATR sampling accessory was used to obtain the spectra of the samples over a scan range of 650–4000 cm^−1^, utilizing 4 scans per sample, and a fixed universal compression force of 85 N.

### 2.4. X-ray Diffraction Spectroscopy

Diffractograms were obtained using a Siemens D500 X-ray powder diffractometer (Karlsruhe, Germany) with Cu Kα radiation (λ = 0.15418 nm). The diffraction was examined in the range of 10° to 80°.

### 2.5. Melt Flow Index

The melt viscosity behavior of polymeric samples was assessed using a Rosand Melt Flow Indexer with a fixed weight of 2.16 kg. Neat polymer materials in powders and extruded forms were processed at 80, 100 and 120 °C, while extruded formulations were processed at 110 °C. The molten material flowed through an orifice of 2.0 mm diameter for 10 min, and the results were reported in g 10 min^−1^.

### 2.6. Preparation of Hot-Melt Extrudates

Hot-melt extrusion was performed on a benchtop Prism™ TSE 16 twin-screw co-rotating extruder with 15:1 length to diameter ratio screws, speed of 50 rpm, and torque in a range of 20–25%. Physical mixtures were fed using an automatic feeder at a rate of 9 g min^−1^ with the temperature from the feeding zone at 83 °C and the barrel at 110 °C.

PEO 95% and Fen 5% (*w*/*w*) were weighed and mixed manually before melt processing. Extrudates were air-cooled and granulated using Colortronic rough granulator machine [14].

Table 1 shows the composition of the formulations developed in this study, in which PEO and Fen were processed, granulated, and processed again adding PCL to minimize the interactions between PCL and Fen that could negatively affect the drug release.

### 2.7. Scanning Electron Microscopy with Energy Dispersive X-ray Spectroscopy

In this case, scanning electron microscopy (TESCAN MIRA) was used for the examination of the topography and morphology of extrudates with energy dispersive X-ray spectrometry (X-Max) for the assessment of their chemical composition and uniformity of the drug distribution. Prior to imaging, samples were attached to an adhesive conducting tape on the stubs and coated with gold particles.

### 2.8. In Vitro Drug Release Measurements

In vitro drug dissolution studies were carried out using a DISTEK dissolution system 2100B. Extruded samples of 5 cm were placed in phosphate buffer media, pH 6.5, 37 ± 0.5 °C, 60 rpm, and 900 mL per vessel. Samples of 4 mL were collected at predetermined time intervals, filtered using PTFE 0.45 μm, and immediately replaced with fresh dissolution media. Fen concentration was measured by UV-Vis spectrophotometer 1280 (Shimadzu) at 288 nm, and calculated using a calibration curve with R^2^ = 0.9993.

## 3. Results and Discussions

### 3.1. Thermal, Physical and Chemical Properties of Extrudates

#### 3.1.1. Thermal Analysis by DSC and TGA/DTG

**Figure 2:**
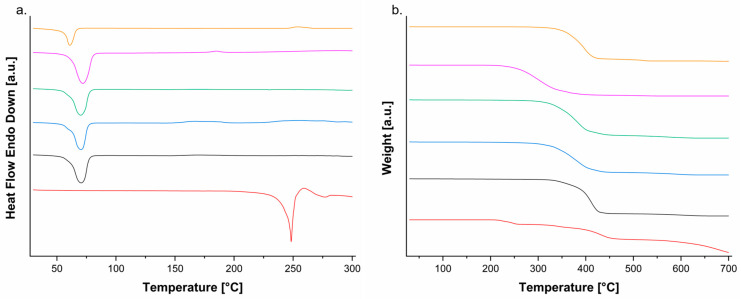
(**a**) DSC and (**b**) TGA curves superposition of Fen (red), formulation 1 (black), formulation 2 (blue), formulation 3 (green), PEO extruded (pink) and PCL extruded (orange) at heating rate of 10 °C min^−1^.

DSC and TGA/DTG tests were carried out to investigate the thermal characteristics of melt-extruded blending of PEO and PCL. Raw materials and extruded formulations were characterized based on their calorimetric (Figure 2a) and thermogravimetric profiles (Figure 2b).

PEO powder is represented by a single endothermic peak characteristic of the crystalline fusion at 66 °C (Δ*H_m_* = 197 J g^−1^), exhibiting this endothermic peak at 68 °C (Δ*H_m_* = 213 J g^−1^) after extrusion. PCL powder is characterized by one single endothermic peak at 57 °C without changing it after extrusion, but the melting enthalpy increased from 69 to 77 J g^−1^. These results correspond with previous studies, which detailed the melting point of neat PEO at 69 °C [11] and neat PCL at 60 °C [18].

Formulations 1, 2 and 3 display one single endothermic melting peak at 67 °C and fusion enthalpy of 190, 182 and 182 J g^−1^, respectively. This endothermic peak is characteristic of PEO melting phase transition since it represents a major part of the formulations, showing subtle energy changes after increasing PCL content. The same result has been reported by Lyons et al. [11], they saw only one melting peak at 65 °C corresponding to the fusion of the PEO/PCL blend and some changes in the peak shape based on the polymers’ ratios.

The miscibility of polymer blends is usually investigated by DSC, where a single glass transition temperature is the criterion applied for determining the miscibility between them. Since the glass transition temperatures of PEO and PCL overlays, the determination of their miscibility by DSC is not viable [5]. In another study using phase contrast microscopy, it was proved the immiscibility of PEO/PCL blends [19]. Nevertheless, the miscibility of PEO and PCL is not a main requisite for their application in drug delivery systems, so it will not be discussed further.

Fen is a crystalline powder represented by two endothermic peaks, one at 244 °C (Δ*H_m_* = 252 J g^−1^) characteristic of the sample melting transition, followed by a second peak at 271 °C (Δ*H_m_* = 27 J g^−1^) representing its decomposition. Other studies have reported the same thermal profile for Fen, exhibiting the melting event followed by decomposition [14,15,20].

HME is a processing technique used to enhance the solubility of low water-soluble drugs by producing solid dispersions. It converts the API’s crystalline structure into its amorphous state and blends both API and polymer chains at the molecular level, due to the high sheer force of the process usually requiring a temperature above the API’s melting point (*T_m_*) [21]. However, studies have proved the possibility of processing solid dispersion formulations below the original melting point of the drug [14,22] since the interactions between drug and polymers may depress the API’s chemical potential, which causes the fusion to take place below the usual *T_m_* [21].

Fen melting peak could not be visualized in the formulations’ thermograms, which can be explained by the capacity of PEO/PCL blends to melt first during melt-processing solubilizing Fen’s crystalline structure, converting it into its amorphous state and creating intermolecular interactions between the drug and polymers [14]. In another study with PEO and etofylline, only the polymer melting peak was evident in the thermogram, so the absence of the API’s phase transition indicates its complete dissolution in the polymer’s amorphous regions [23]. Whilst, Hurley et al. [8] working with solid dispersion formulations of indomethacin reported the complete disappearance of its melting peak, indicating that the drug was entirely converted to its amorphous state, describing their solid dispersion as a two-phase system due to the presence of the peak of the semi-crystalline polymer, as we have seen in our study with Fen converted to its amorphous state and the polymers in their semi-crystalline form.

We can assume that the noticeable melting point depression of Fen reveals its good miscibility with PEO/PCL blend, which can be attributed to intermolecular interactions between them. For a drug and polymer to be miscible, it requires the drug to be successfully converted to its amorphous form interacting with the polymeric carrier [8] as we have seen in our study processing and melting Fen below its melting point.

TGA/DTG analysis is a common method for studying the polymer’s degradation process. Neat PEO was stable below 193 °C with two stages of mass loss. The first stage from 193 to 412 °C (∆*_m_* = 96%) with T*_peak_* DTG = 306 °C and the second one from 413 to 560 °C (∆*_m_* = 2%) with T*_peak_* DTG = 519 °C. After extrusion, neat PEO demonstrated a very similar degradation profile when compared to the powder form, but its stability decreased to 178 °C. While neat PCL was stable below 263 °C with two stages of decomposition. One stage from 263 to 472 °C (∆*_m_* = 92%) with T*_peak_* DTG = 409 °C, and the second stage from 473 to 554 °C (∆*_m_* = 6%) with T*_peak_* DTG = 514 °C. After extrusion, neat PCL reproduced a very similar behavior to its powder state, but the stability was reduced to 257 °C. This decrease in the thermal stability of both polymers after extrusion can be linked to their molecular weight variations as a consequence of exposure to thermal and mechanical stresses during melt-extrusion [24]. For example, melt fracture may occur when the polymer chains are forced to organize themselves to pass through the die recoiling into a random configuration upon exit, leading to increased chain scission [25].

Fen was stable below 164 °C followed by four stages of decomposition. The first stage from 164 to 269 °C (∆*_m_* = 13%) with T*_peak_* DTG = 241 °C attributed to the loss of sulphur atom, the second was 270 to 357 °C (∆*_m_* = 8%) with T*_peak_* DTG = 334 °C represented by the loss of CH_3_O, the third was 358 to 506 °C (∆*_m_* = 34%) with T*_peak_* DTG = 428 °C associated to the loss of C_6_H_5_ and CO molecules, and the last one was 507 to 700 °C (∆*_m_* = 34%) with T*_peak_* DTG = 672 °C represented by the loss of C_7_H_5_N_3_ molecule. The difference in mass loss (∆*_m_* = 11%) corresponds to the carbonaceous residue. Similar results have been described in the literature [26].

Formulations 1, 2 and 3 displayed two stages of decomposition with stability below 287, 203 and 266 °C, respectively. The first stages from 287 to 463 °C (∆*_m_* = 91%) with T*_peak_* DTG = 405 °C; 203 to 461 °C (∆*_m_* = 91%) with T*_peak_* DTG = 374 °C and 266 to 474 °C (∆*_m_* = 90%) with T*_peak_* DTG = 375 °C followed by the second stages from 464 to 663 °C (∆*_m_* = 6%) with T*_peak_* DTG = 597 °C; 462 to 660 °C (∆*_m_* = 7%) with T*_peak_* DTG = 587 °C and 475 to 643 °C (∆*_m_* = 7%) with T*_peak_* DTG = 577 °C, respectively. Despite being a major part of these formulations, PEO once blended with PCL has shown to increase the drug thermal stability within the melt-extruded matrices starting their decomposition process at over 200 °C.

#### 3.1.2. Physical and Chemical Evaluation by FTIR and XRD

FTIR is an efficient technique for the investigation of interactions between the API and polymers, which are required for drug-polymer miscibility, through the definition of their chemical structure and molecular conformation by the absorption bands in the regions of the infrared spectrum [21].

**Figure 3:**
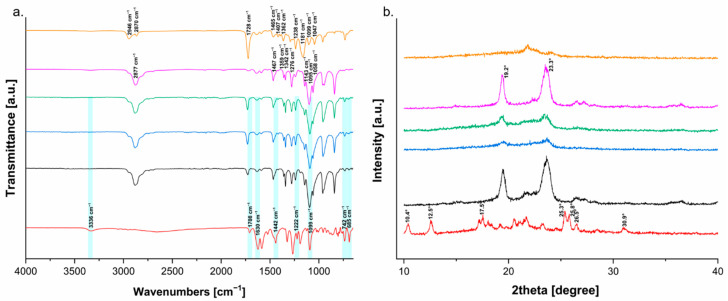
(**a**) FTIR spectra and (**b**) XRD diffraction patterns of Fen (red), formulation 1 (black), formulation 2 (blue), formulation 3 (green), PEO extruded (pink) and PCL extruded (orange).

Figure 3a displays the IR spectra corresponding to raw materials and melt-extruded formulations. Fen spectrum was confirmed by comparison with SpectraBase^TM^/Wiley (CAS #43210-67-9), identifying its relevant signals at 3336 cm^−1^ attributed to stretching mode of (N-H) from the carbamate group, 1708 cm^−1^ attributed to the (C=O) stretching vibrations of the carbamate carbonyl, 1630 cm^−1^ attributed to the (N-H) bending and the (C-N) stretching modes, 1442 cm^−1^ assigned to the (C-N), 1222 cm^−1^ attributed to the (C-O), 1099 cm^−1^ attributed to the phenyl-(o), 742 cm^−1^ characteristic of phenyl group, and 685 cm^−1^ identified as benzenethiol moiety [18,26,27,28].

PEO is spectroscopically characterized by a majority of its peaks attributed to methylene groups at 2877 cm^−1^ symmetric stretching, 1467 cm^−1^ scissoring, 1359 and 1342 cm^−1^ wagging, and 1276 cm^−1^ twisting. Moreover, the presence of (C-O-C) as a triple peak at 1143, 1095 and 1058 cm^−1^ stretching reflects its semi-crystalline phase [29,30]. Whilst PCL spectrum is expressed by methylene group vibrations at 2946 and 2870 cm^−1^, a sharp intense peak at 1728 cm^−1^ characteristic of (C=O), signals at 1465, 1407 and 1362 cm^−1^ characteristic of (CH_2_) bending, 1238 and 1181 cm^−1^ attributed to (COO), 1099 and 1047 cm^−1^ related to (C-O) vibrations [31].

When the neat Fen spectrum is compared to the spectra of formulations 1, 2 and 3, it is not seen any new peak that could indicate a chemical interaction between the API and polymers. However, it is evident the disappearance of the band at 3336 cm^−1^, dislocation of the signal from 1708 to 1732 cm^−1^, expressive reduction of the band at 1630 cm^−1^, dislocation of the signal from 1442 to 1465 cm^−1^, followed by expressive reduction of peaks’ intensity at 742 cm^−1^ and 685 cm^−1^. The dislocation and reduction of signals characteristic of Fen when mixed with PEO/PCL blends implies a decrease in the drug’s degree of crystallinity due to changing into its amorphous state and establishing intermolecular interactions [30]. These interactions can be endorsed by a reduction of the signal at 1276 cm^−1^ attributed to the methylene group of PEO interacting with Fen through hydrophobic interactions, being PEO hydrophobic properties reported by other studies [14,30]. Moreover, a reduction and dislocation of the band at 1728 cm^−1^ (C=O), and the disappearance of the signals at 1181 cm^−1^ (COO) and 1047 cm^−1^ (C-O) from PCL can be interpreted as physical interactions by hydrogen bonding with the polar groups present in the molecule of the API [14]. These findings support our calorimetric data, demonstrating Fen has good miscibility with PEO/PCL blends, which is vital for the successful conversion of the API crystalline content into its amorphous form.

XRD was applied in this study to investigate the amorphous nature of the API inside the melt-extruded matrices. Figure 3b shows the results obtained from the Fen diffractogram revealing several peaks well-evidenced, which indicates the crystalline nature of the drug. The main crystalline peaks occurred at diffraction angles of 10.40°, 12.59°, 17.57°, 25.31°, 25.81°, 26.50°, and 30.98° with a similar result described in the literature [20].

**Figure 4:**
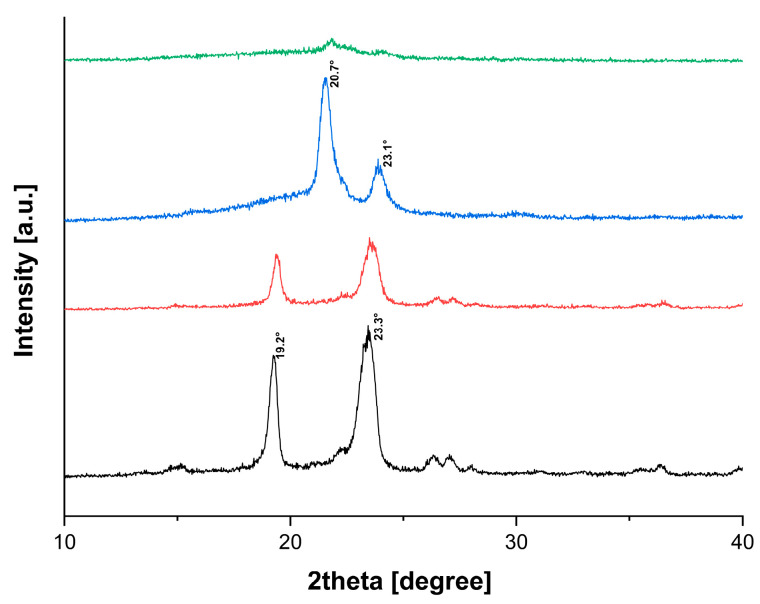
XRD shows the diffraction peaks of PEO powder (black), PEO extrudate (red), PCL powder (blue) and PCL extrudate (green).

After analysing the diffractogram of each neat polymeric excipient before and after extrusion (Figure 4), it was seen that the two principal diffraction peaks of PEO occurring at 19.2° and 23.3° [32] decreased after extrusion processing. The same phenomena happened to PCL, which exhibited two diffraction peaks at 20.70° and 23.19° [33], but they almost disappeared as a consequence of the processing. These data confirmed the reduction of PEO and PCL crystallinity content after the processing by HME.

XRD analysis of extruded formulations 1, 2 and 3 (Figure 3b) confirmed the amorphous nature of Fen within the monolithic matrices of PEO and PCL since the main drug diffraction peaks associated with the drug crystallinity were completely absent. This information can support the hypotheses raised in the calorimetric study that our solid dispersion is a two-phase system where Fen and PCL were successfully converted to their amorphous state, while PEO remains semi-crystalline.

### 3.2. Material Melt Viscosity by MFI

The MFI is an easy measurement method responsible for providing rheological information that is required to facilitate the processing of polymers using techniques such as extrusion and injection molding [34]. Due to its thermoplastic behavior, PEO has been extensively applied to the development of pharmaceutical formulations using HME. However, the combination of different PEO molecular weights and/or other polymers such as PCL are required to not only tailor the drug release profile but also to enhance the melt-extrusion process [35].

**Figure 5:**
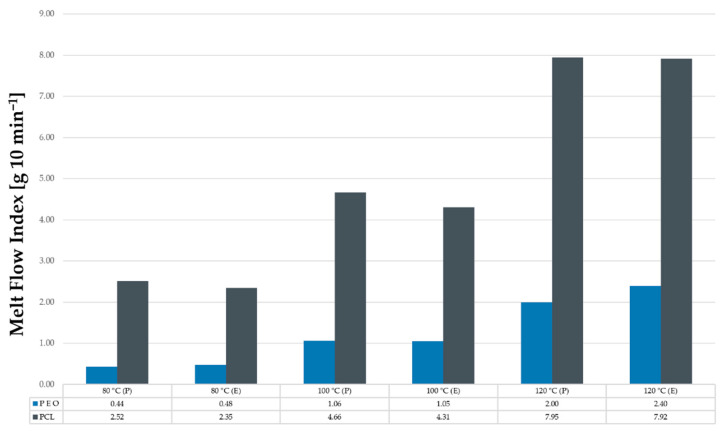
Melt flow index readings for P = powder and E = extrudate of PEO and PCL at 80, 100 and 120 °C.

MFI test was carried out on neat PEO and neat PCL before and after extrusion at 80, 100 and 120 °C to investigate the thermal and mechanical effect caused by the melt processing on their flow properties. In Figure 5, it is seen that PEO has low melt viscosities than PCL, but improved from 0.44 g 10 min^−1^ (P) and 0.48 g 10 min^−1^ (E) to 2 g 10 min^−1^ (P) and 2.4 g 10 min^−1^ (E) as we increased the temperature from 80 to 120 °C. Lyons et al. [11] reported the addition of a less viscous polymer such as PCL to enable more viscous polymers such as PEO to be melt processed easily.

PCL melt viscosities increased substantially from 2.52 g 10 min^−1^ (P) and 2.35 g 10 min^−1^ (E) to 7.95 g 10 min^−1^ (P) and 7.92 g 10 min^−1^ (E) as the temperature increased from 80 to 120 °C. Grehan et al. [5] reported that increasing the content of PCL in a formulation leads to a higher melt flow index, causing a decrease in the viscosity, and, consequently, a better flow.

Additionally, the polymers’ thermal stability is crucial for the development of monolithic matrices with extended-release profiles, so the study of critical variables such as melt viscosity is crucial for the determination of the HME processing temperatures in order to produce thermal stable extruded matrices [35]. Based on this information, our formulations were melt-processed at 110 °C. MFI measurements of extrude formulations 1, 2 and 3 were also performed at 110 °C displaying melt viscosities of 2.68, 2.82 and 2.86 g 10 min^−1^, respectively. In other words, PCL acts as a plasticiser when melt-processed with PEO, increasing the MFI, which leads to a better flow and processing properties of the polymeric matrices.

### 3.3. Drug Dispersion Assessment by SEM and EDX

SEM is a high-level microscope technique where an energetic and focused electron beam scans the sample providing information about its topography, morphology and composition. Additionally, the combination of SEM imaging with EDX spectra turns it into one of the utmost versatile tools in scientific research [36].

**Figure 6:**
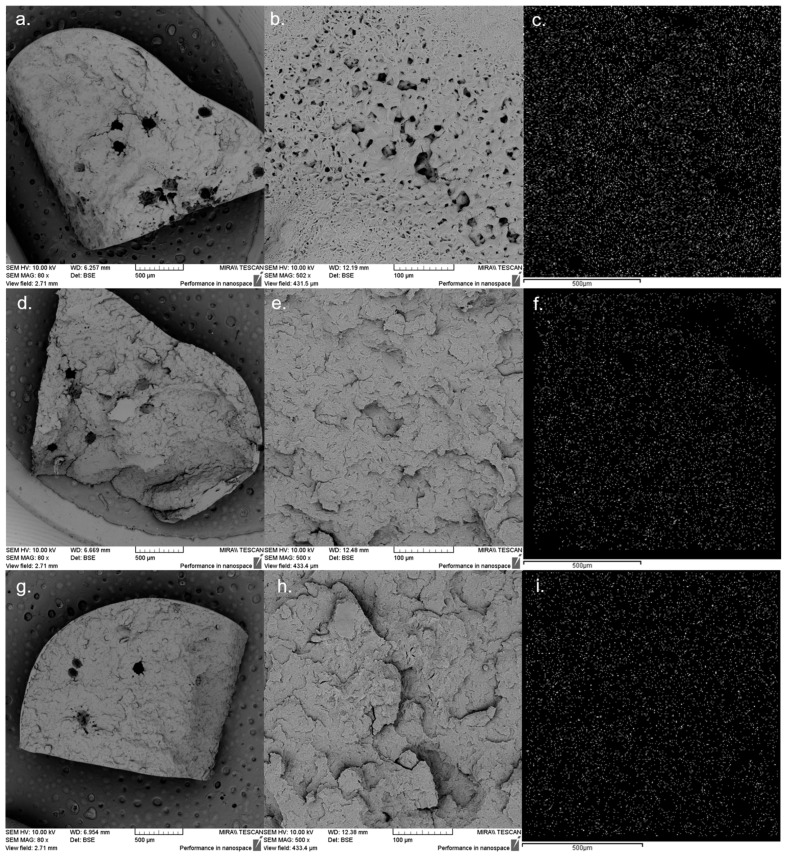
SEM images of formulation 1 (**a**,**b**), formulation 2 (**d**,**e**) and formulation 3 (**g**,**h**) showing the topography and morphology of extrudates followed by EDX analysis of formulation 1 (**c**), formulation 2 (**f**) and formulation 3 (**i**) showing the homogeneous distribution of the element sulfur from Fen within PEO/PCL blends.

In Figure 6a,b are seen the scanning electron micrographs of formulation 1, Figure 6d,e of formulation 2 and Figure 6g,h of formulation 3. All formulations display irregular surfaces with fissures, voids and pores of different sizes and shapes connected through ducts forming a dense network that can slower the drug release [37].

The drug dispersion inside the polymeric matrix was assessed by EDX for formulations 1 (Figure 6c), formulation 2 (Figure 6f) and formulation 3 (Figure 6i). The molecular formula of Fen is C_15_H_13_N_3_O_2_S [38], while the PEO/PCL blend-based matrices are basically constituted by carbon, oxygen and hydrogen. The EDX spectra of all three formulations found that carbon, oxygen and sulfur are the most common elements among the samples, the latter being, specifically, exclusive of the API. Thus, a filter was applied to map only the sulfur atom characteristic of the drug dispersed within the melt-extruded matrices, being clearly evident through the bright spots the homogeneous distribution of Fen throughout the polymeric carrier.

The literature states that increasing some processing parameters during melt extrusion, such as the barrel temperature, can lead to lower free volume, smaller pore radius, and a more complex pore network, causing the drug release rates to reduce [37]. Since our formulations had been subjected to additional shear and heat from being extruded twice, we expect this second processing stage can also have some effect on slowing the drug release rate, which requires further evidence obtained from in vitro dissolution studies.

### 3.4. Drug Release from Extrudates

The literature outlines that a drug release pattern can be modulated via different release mechanisms, which can be affected by the polymeric carrier, the drug properties and the environmental conditions [39].

The objective of this study was to extend the drug release profile of Fen using PEO/PCL blend-based matrices. PEO is a hydrophilic polymer with the main release mechanism classified as anomalous, which means it is driven by swelling, drug diffusion and matrix erosion, with faster drug release rates influenced by higher PEO concentrations [37]. Whilst PCL is a hydrophobic polymer responsible for slowing the drug release rates from a polymeric matrix due to reducing the penetration of water molecules into the system [40]. In our previous work [14], a solid dispersion formulation of PEO 95% and Fen 5% (*w*/*w*) was able to release completely the drug within 3 h. Thus, we expected that inserting PCL in our formulations to reduce the release rate of Fen.

**Figure 7:**
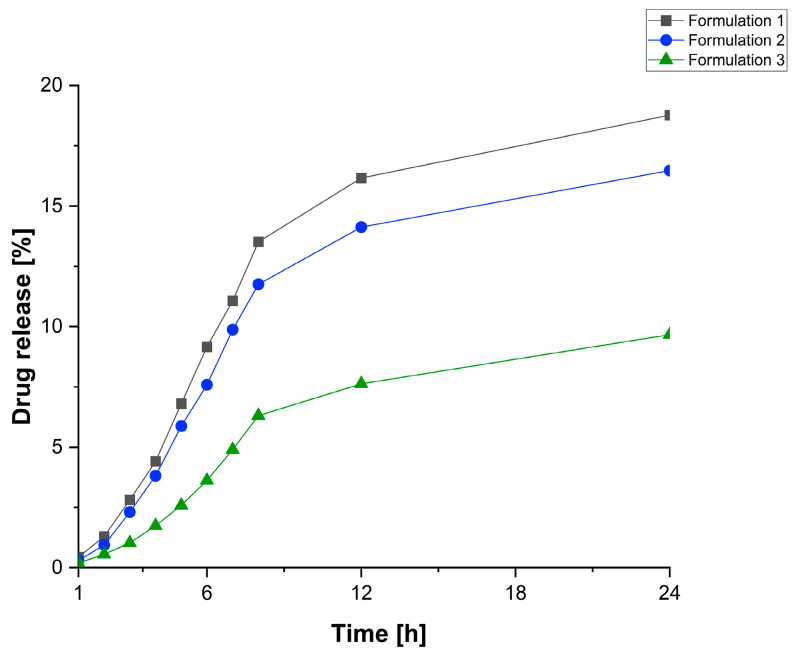
Cumulative release percentage of Fen from formulation 1, formulation 2 and formulation 3 in phosphate buffer media.

Figure 7 shows the dissolution profile of our solid dispersion formulations comprising Fen, PEO and different amounts of PCL, presented as a cumulative release percentage. All three formulations displayed a similar profile as they increased the first stage of drug release to up to 8 h. Formulations 1, 2 and 3 released 13%, 11% and 6% of the drug, respectively, followed by a sustained release behavior over 24 h achieving 18%, 16% and 9%. Lyons et al. [11] described that PCL retards the drug release from monolithic matrices proportionally to the content of such polymer incorporated in the matrix, which supports our findings.

Moreover, all three extruded formulations revealed a two-stage drug release profile consisting of an immediate release followed by a sustained release, which helps to overcome the issues related to a multiple-dosing regimen. A similar profile was described by Hassan et al. [41] working with curcumin as their formulation displayed a fast release of 18% over one hour, followed by a sustained release of 22.3% during the remaining hours. Sanna et al. [42] developing controlled-release systems for ruminants applied PCL and polymethylmethacrilates for the oral administration of folic acid, they also found the same drug release pattern characterized by a biphasic behavior with an initial quick release of folic acid followed by a plateau. Whilst, Pepic et al. [10] working on the release behaviour of carbamazepine-loaded PCL/PEO microspheres reported an initial burst followed by a steady increase of the drug concentration during the whole period of study.

Since the 1980s, drug delivery systems have been marketed to provide veterinarians and farmers with means to control parasitic diseases in ruminants [16]. However, the development of new treatments for these animals has been challenging due to the complexity of their gastrointestinal tract; for instance, the reticulo-rumen pH ranges between 5 and 7 and the intestinal pH increases gradually from 2.7 to 7.5 [43], becoming even more arduous to establish a standard in vitro dissolution testing for new formulations [44]. Although these extruded formulations would not appear useful for commercial extended-release systems based on the drug release results achieved under the conditions applied in our dissolution study, the incorporation of a hydrophobic polymer such as PCL into a hydrophilic matrix of PEO processed by HME has proved to be an attractive option to retard the release rate of Fen.

## 4. Conclusions

This study highlighted the feasibility of HME technology in the development of extended-release extruded formulations of Fen dispersed in PEO/PCL blend-based matrices.

Thermal analysis of extruded formulations displayed one single endothermic melting peak characteristic of the PEO melting phase, while the absence of Fen melting peak can be explained by its conversion to an amorphous state, which is characteristic of a two-phase solid dispersion system. Moreover, the melting point depression of Fen made clear its good miscibility with PEO/PCL blend matrices, which also revealed to increase the drug’s thermal stability. FTIR analysis of the formulations exhibited dislocation and reduction of some main peaks of the API, representing a decrease in its crystallinity and changing to an amorphous state responsible for establishing intermolecular interactions with the polymeric matrix. XRD analysis of the formulations confirmed the amorphous nature of Fen within the monolithic matrices, endorsing our solid dispersion as a two-phase system. MFI test revealed that PCL acts as a plasticizer when processed with PEO, leading to better flow properties and contributing to easier material processing. SEM images showed that the melt-extruded matrices had irregular surfaces with fissures, voids and pores of different sizes and shapes forming a dense network responsible for slowing the drug release, whilst EDX spectra demonstrated a homogeneous drug distribution throughout the polymeric carrier. Dissolution testing showed that PCL retards the drug release from monolithic matrices proportionally to the content of such polymer incorporated. All formulations revealed similar drug delivery profiles with a fast release over 8 h, followed by a sustained release over 24 h, which is characteristic of a biphasic system.

Thus, the melt-extruded polymeric matrices developed in this study showed that the drug release rate in a PEO/PCL blend can easily be tailored by altering the ratio of PCL to address the issues related to the multiple-dosing regimen of Fen, and, consequently, enhancing the treatment efficacy in ruminants.

## Figures and Tables

**Table 1:** Description of the formulations’ composition.

Composition	Formulation 1	Formulation 2	Formulation 3
PEO + Fen	90%	80%	70%
PCL	10%	20%	30%

## Data Availability

Not applicable.
